# Evolutionary Dynamics of Tat in HIV-1 Subtypes B and C

**DOI:** 10.1371/journal.pone.0129896

**Published:** 2015-06-18

**Authors:** Chandra Nath Roy, Irona Khandaker, Hitoshi Oshitani

**Affiliations:** Department of Virology, Tohoku University Graduate School of Medicine, 2–1 Seiryou machi, Aoba-ku, Sendai City, Japan; National Institute of Health, ITALY

## Abstract

Evolutionary characteristics of HIV-1 have mostly studied focusing its structural genes, Gag, Pol and Env. However, regarding the process of HIV-1's evolution, few studies emphasize on genetic changes in regulatory proteins. Here we investigate the evolutionary dynamics of HIV-1, targeting one of its important regulatory proteins, Tat. We performed a phylogenetic analysis and employed a Bayesian coalescent-based approach using the BEAST package to investigate the evolutionary changes in Tat over time in the process of HIV-1 evolution. HIV-1 sequences of subtypes B and C from different parts of the world were obtained from the Los Alamos database. The mean estimated nucleotide substitution rates for Tat in HIV-1 subtypes B and C were 1.53x10^-3^ (95% highest probability density- HPD Interval: 1.09 x10^-3^ to 2.08x10^-3^) and 2.14x10^-3^ (95% HPD Interval: 1.35 x10^-3^ to 2.91x10^-3^) per site per year, respectively, which is relatively low compared to structural proteins. The median times of the most recent common ancestors (tMRCA) were estimated to be around 1933 (95% HPD, 1907–1952) and 1956 (95% HPD, 1934–1970) for subtypes B and C, respectively. Our analysis shows that subtype C appeared in the global population two decades after the introduction of subtype B. A Gaussian Markov random field (GMRF) skyride coalescent analysis demonstrates that the early expansion rate of subtype B was quite high, rapidly progressing during the 1960s and 1970s to the early 1990s, after which the rate increased up to the 2010s. In contrast, HIV-1 subtype C exhibited a relatively slow occurrence rate until the late 1980s when there was a sharp increase up to the end of 1990s; thereafter, the rate of occurrence gradually slowed. Our study highlights the importance of examining the internal/regulatory genes of HIV-1 to understand its complete evolutionary dynamics. The study results will therefore contribute to better understanding of HIV-1 evolution.

## Introduction

Since it was first detected in 1981, the HIV-1 virus has shown high genetic variability, generating highly divergent strains. The transmission of those HIV-1 variants is a dynamic process because of continuous intermixing among different strains due to increasing human mobility across the globe. From the HIV-1 virus' perspective, diversity in the genome significantly impacts the functionality and immunogenicity of the virus itself, and this diversity is estimated to be about 1% per year [1,2].

The possible reasons for such divergence are high error rate during reverse transcription [3] and increased virus production [4] due to comparatively fast replication kinetics [5]. On the other hand, HIV-1 rapidly evolves during the course of infection in response to selective pressure induced by the host's immune response [6]. Therefore, to develop an effective vaccine, it is necessary to continuously monitor rapid genetic changes [7,8]. The evolution of structural genes in HIV-1 disease progression has been studied; there were several studies focused on molecular evolution in subtypes B and C in different high-risk groups targeting Gag, Pol and Env genes [9–14]. However, the diversity and evolution of regulatory proteins like Tat are yet to be elucidated. Tat is known as an important factor in disease progression as it transactivates viral transcription, which plays a central role in the regulation of HIV-1 gene expression. A previous study shows that Tat sequence diversity among patients infected with HIV-1 subtype B prompted different speeds of disease progression [9]. However, the evolution of HIV-1 Tat has yet to be studied. The aim of the current study is therefore to observe evolutionary changes over time in non-structural HIV-1 genes like Tat. To this end, here we our goal is to report the dating, substitution rate, and demographic dynamic of Tat in major HIV-1 subtypes like B and C as these two subtypes account for about 60% of the total HIV-1 infected cases distributing worldwide [15].

## Materials and Method

### Nucleotide sequence dataset

We curated the full- length HIV-1 Tat sequences from the ‘Web alignment’ set of the Los Alamos National Laboratory (LANL)'s online HIV sequence database (http://www.hiv.lanl.gov/content/sequence/NEWALIGN/align.html) [16] using options as follows: “Alignment type: Web (all complete sequence), Year: 2012, Organism: HIV-1, Region: TAT, Subtype: All, DNA/Protein: DNA, Format: FASTA” accessed on February 2, 2014. A total of 2156 sequences were downloaded preliminary and after excluding the sequence with no date, the numbers of sequences were reduced in to 2058. We included ‘one sequence per patient’ using in-built search interface of Los Alamos National Laboratory (LANL) web alignments. According to LANL, sequences for ‘web alignment’ were sorted alpha-numerically by accession in case of multiple deposited sequences of a single patient, therefore, the selected sequence should be the sequence having first accession number in alpha-numeric order the sequence; however, it may not always the earliest sample. We also excluded problematic sequences using LANL'S pre-built search options. As defined by the LANL, problematic sequences mean high content of non-ACTG characters, probable contamination with a laboratory strain, G->A hypermutation, synthetic sequences, sequence containing an artifactual deletion of >100 nucleotides, tiny sequence (< 50 basepairs), sequence that was deposited as its reverse complement, percentage of non-ACGT characters in the nucleotide sequence etc. Through this process, a total of 713 sequences for subtype B and 353 sequences for subtype C, were obtained. The sequences were then divided into two data sets based on HIV-1 subtypes B and C. The reference strains for each subtype were also downloaded from the ‘Subtype Reference Alignment’ set. We then submitted all the sequences to the Recombinant Identification Program (RIP) (http://www.hiv.lanl.gov/content/sequence/RIP/RIP.html, accessed on March 28, 2014)[17] to eliminate the eventual intersubtype recombinants sequences. Furthermore, ambiguous nucleotides, hypermutation, minor insertions, minor deletions, premature termination codons, and different sequences from the same strains were also excluded after checking every sequence. In order to better determine and visualize the relationship between sequences from different geographic areas, we attempted to include sequences from every country for both subtypes. We finally prepared a dataset of 135 and 53 sequences for subtypes B and subtype C, respectively. The datasets contained sequences from a total of 45 countries from six different continents: Africa (8 countries), North America (5 countries), South America (11countries), Asia (9 countries), Oceania (1 country), and Europe (11 countries). These sequences contained 100 amino acids from 300 nucleotide (nt) positions, 5831 to 6045nt in exon 1 and 8379 to 8463nt in exon 2, in the consensus sequence of the HIV-1_HXB2_ genome (GenBank accession# K03455). The sequences were obtained together with information about the host, subtype, isolation year, and isolated country (S1 Table).

### Sequence alignment and phylogenetic tree analysis

The nucleotide sequences (with no gap) were aligned by using ClustalW: a multiple-sequence alignment program. We then used Xia’s test implemented in DAMBE to examine the sequences saturation which is useful for the phylogenetic analysis (Phylogenetic signal) [18–20]. Maximum Likelihood and Neighbor-Joining phylogenies were inferred using the GTR+I+C5 of nucleotide substitution model with gamma distribution (Γ) and a proportion of invariable sites (I), as it was selected one of the best models in both jModelTest 2.1.6 [21] and Molecular Evolutionary Genetic Analysis (MEGA) version 6 [22]. Positions containing gaps and missing data were excluded. Reliability was estimated from 1,000 bootstrap replicates (Figures A and B in S1 Fig). The CPZ.US.85.US_Marilyn.AF103 strain was used as an out-group.

### Estimation of evolutionary rates and dates

The substitution rate and timing of the most recent common ancestor (tMRCA) of subtypes C and B were estimated by BEAST v1.8.1 [23].The GTR+Γ+I substitution model was used in all runs. Following the procedure described in the previous study [10], we did BEAST analysis using normal distribution with a mean of 2.5x10^-3^ and standard deviation 5x10^-4^ prior using three different molecular clock models: strict relaxed, uncorrelated exponential, and relaxed uncorrelated lognormal [24,25]. Strength of model selection was assessed with a Bayes factor (BF) test with the Akaike information criterion-Monte Carlo (AICM) [26] in Tracer v.1.6. The results showed that the strict clock model was best for both subtype C and B. With sub-sampling performed every 2.56105 steps, MCMC simulations were run for 2.56108 chain steps and analyzed by Tracer. For each tested prior, we considered a value above 250 to be an effective sample size (ESS). The Maximum Clade Credibility (MCC) tree, with time scale, was created using Tree Annotator v1.8.1 with a burn-in of the first hundred trees and illustrated using Fig tree version 1.4.2 [27].

### Demographic dynamics

We did phylodynamic analysis of the HIV-1 virus using the Tat to understand the trend of virus evolution over time. The phylodynamic histories of HIV-1 subtypes B and C were estimated from the Tat gene sequences using Bayesian skyride, which is an extension of the Bayesian Skyline Plot (BSP) implemented in BEAST. In Bayesian skyride analysis, changes in population size between the intervals are smoothed, and thus, we could assume gradual changes in population size using the time-ware prior [25]. Notably, Gaussian Markov random field (GMRF) smoothing prior is used in Bayesian skyride analysis tool. A marginal posterior distribution of the demographic inference was estimated using the Bayesian MCMC method and the effective population size at the most recent time of sampling as well. We analyzed those posterior samples using the Tracer program.

## Results

### Estimation of evolutionary characteristics HIV-1 Tat

#### Substitution rate

The mean estimated substitution rates for subtypes B and C were 1.53x10^-3^ (95% HPD Interval: 1.09 x10^-3^ to 2.08x10^-3^) and 2.14x10^-3^ (95% HPD Interval: 1.35 x10^-3^ to 2.91x10^-3^) per site per year, respectively. The mean substitution rate was lower in subtype B than subtype C according to analyses using all three models (Table 1). Notably, evolutionary potential of a population is expressed by evolutionary rate, which indicates the number of mutations that are fixed per unit of time in the virus population.

**Table 1:** Substitution rates and time of the most recent common ancestors (tMRCAs) of subtypes B and C for all tested molecular clock methods.

Subtype	Prior	Molecular clock	Marginal likelihood	AICM	Bayes Factor(vs. Lognormal)	Substitution rate	95% HPD		tMRCA			
						Mean	Lower	Upper	Median	Mean	95% HPD	
											Lower	Upper
B	N [2.5x10^-3^, 5x10^-4^]	Strict	-8861.6551	18382.751	63.282	1.53E-03	1.03E-03	2.08E-03	1933	1931	1907	1952
		Exponential	-8910.0671	18404.112	41.921	1.50E-03	6.57E-04	2.53E-03	1917	1907	1837	1958
		Lognormal	-8866.5294	18446.033	-	1.49E-03	8.59E-04	2.08E-03	1931	1928	1896	1956
C	N [2.5x10^-3^, 5x10^-4^]	Strict	-3641.8741	7479.63	10.648	2.14E-03	1.35E-03	2.91E-03	1956	1954	1934	1970
		Exponential	-3636.6521	7506.352	-26.721	2.13E-03	1.25E-03	3.05E-03	1946	1942	1911	1967
		Lognormal	-3638.9247	7468.983	-	2.14E-03	1.39E-03	2.88E-03	1955	1954	1935	1971

### Molecular dating analysis

Bayesian MCMC methodology was used to calculate the time of the most recent common ancestors (tMRCAs) of the sequences of subtypes B and C taken from the global sequences available in the Los Alamos database. The median dates of tMRCAs were estimated to be around 1933 (95% HPD, 1907 to 1952) and 1956 (95% HPD, 1934 to1970) for subtypes B and C, respectively, with a Bayesian model. According to our analysis, subtype C appeared in the global population two decades after the introduction of subtype B.

**Fig 1:**
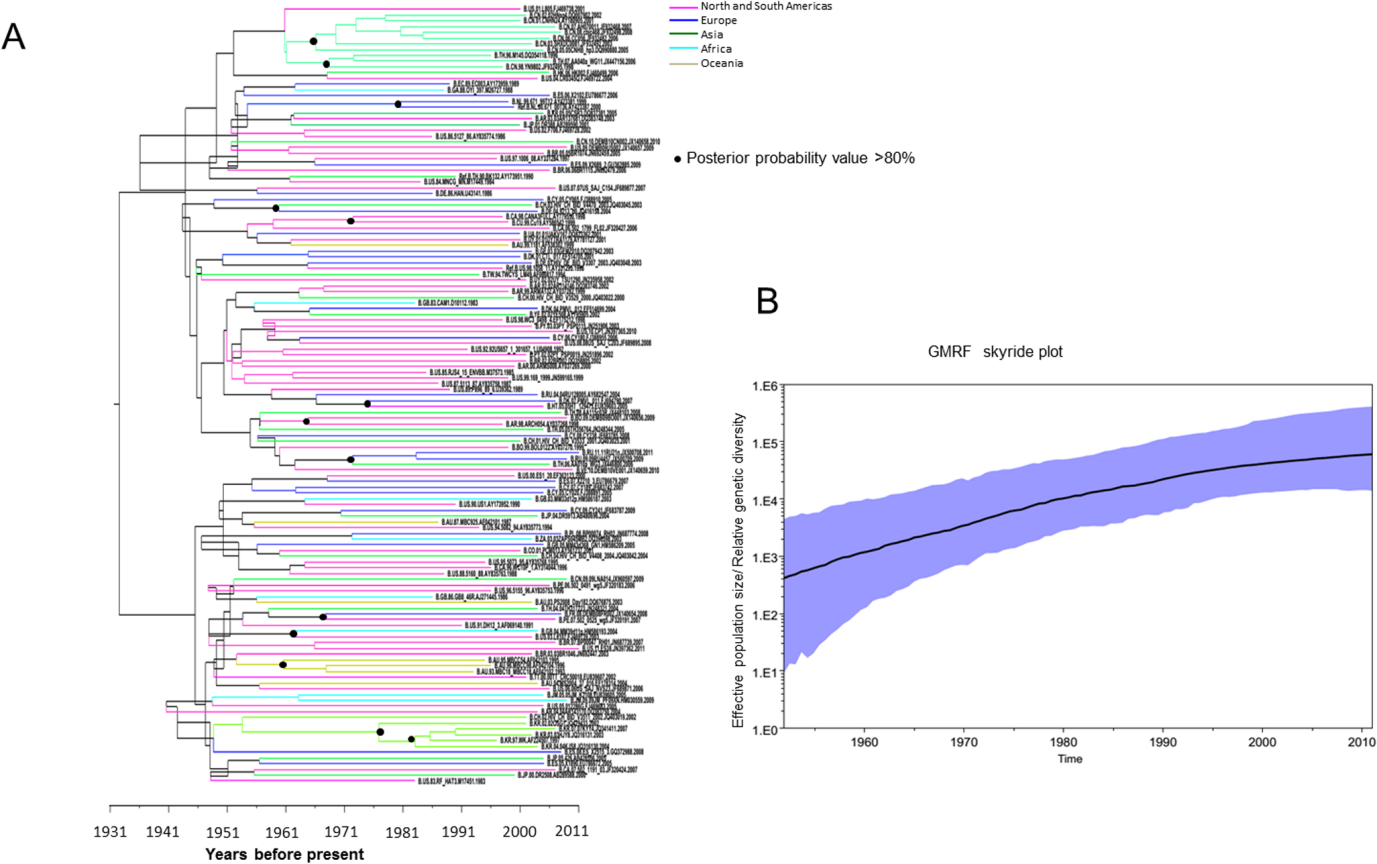
Phylodynamic and Bayesian tree with timescale of HIV-1subtype B Tat sequences from Los Alamos Database. A) Maximum clade credibility tree with time scale obtained from the strict molecular clock. Time to the most recent common ancestors (tMRCA) was indicated in years at the bottom of the figure. B) Gaussian Markov random field (GMRF) skyride plot estimated by strict clock method. The X-axis represents the time in year. The Y-axis represents the HIV-1 Tat effective number of infections (genetic diversity). The black line marks the median estimate for effective population size and the blue shading shows region displays the 95% highest posterior density (HPD) interval.

**Fig 2:**
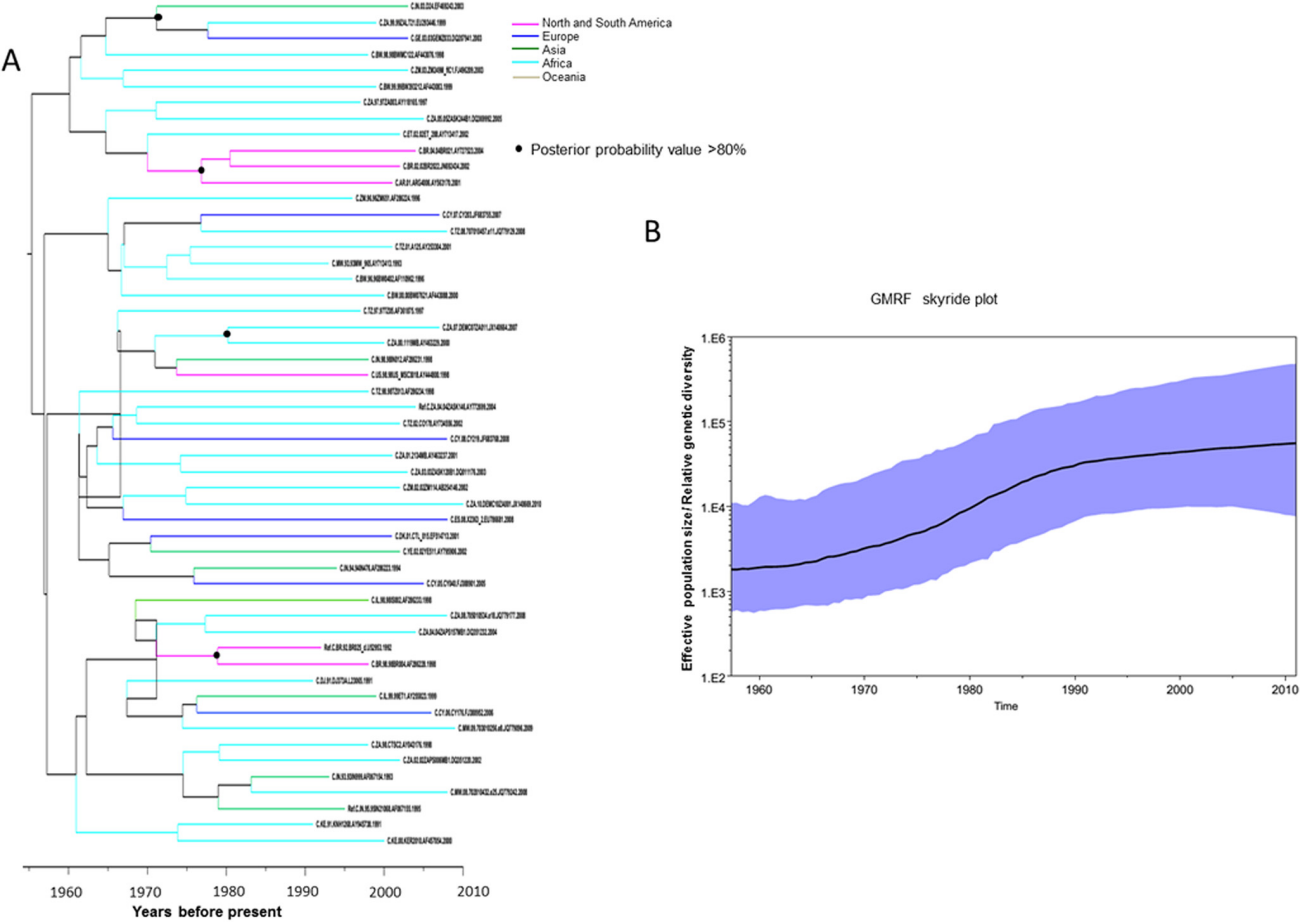
Phylodynamic and Bayesian tree with timescale of HIV-1subtype C Tat. **A) Maximum clade credibility tree with time scale obtained from the strict molecular clock.** Time to the most recent common ancestors (tMRCA) was indicated in years at the bottom of the figure. B) Gaussian Markov random field (GMRF) skyride plot estimated by strict clock method. The X-axis represents the time in year. The Y-axis represents the HIV-1 tat effective number of infections (genetic diversity). The black line marks the median estimate for effective population size and the blue shading showed region displays the 95% highest posterior density (HPD) interval.

In the case of subtype B, South American strains were introduced around the 1940s, whereas most of the North American strains appeared around the middle of 1950s. Asian strains evolved in the middle of the 1940s; strains originating from China, exhibited two peaks: one was from the middle of the 1960s to the middle of the 1970s, and another was from the middle of the 1980s to the middle of the 1990s. African strains mainly evolved around the middle of 1970s. European strains were introduced at various times, ranging from the early 1950s to the early 1980s (Fig 1A). In the case of subtype C, African strains were introduced in the middle of the 1960s; however, most of these strains emerged during the 1970s. Asian strains were first introduced in the early 1970s. European strains emerged earlier than Asian strains, and American strains emerged around the late 1970s (Fig 2A).

### Phylodynamic analysis

Temporal changes in genetic diversity (population size) were observed using a GMRF skyride coalescent model. This analysis demonstrates that the early expansion of subtype B was quite rapid, but the duration was relatively short with a dispersion occurring during the late 1980s. There was an exponential escalation in the occurrence of infections from 1960–1970 to the early 1990s with an increasing trend up to the 2010s (Fig 1B). The demographic history of HIV-1 C exhibited a relatively slow occurrence rate until the late 1980s, and then, there was a sharp increase in occurrences up to the end of the 1990s. Thereafter, the rate of occurrence has gradually slowed (Fig 2B).

## Discussion

In this study, we investigated the evolutionary dynamics and molecular dating of HIV-1 from the perspective of one of its regulatory proteins, Tat. Previously, HIV-1 evolutionary analyses were mainly performed on the virus' structural proteins, Env, Gag and Pol; however, updated genetic information targeting regulatory protein like Tat is limited. We have recently reported the genetic variation of HIV-1 Tat exon 1 in different subtypes and full-length Tat in subtype B and C at global level [28,29]. In our current study, we estimated that nucleotide substitution rates for Tat in HIV-1 subtype B and C were relatively low. Previous studies have shown that the estimated HIV-1 evolutionary rate was 1 to 17 x10^-3^ substitutions per site per year [11,30]. To the best of our knowledge, this is the first report demonstrating substitution rates for the Tat gene in major HIV-1 subtypes. As reported previously, nucleotide substitution rates for the HIV-1 surface glycoprotein, Env, were within the range of 7.3–8.7 x10^-3^substitutions per site per year [11,30]. Notably, HIV-1 subtype B strains from both North America and Europe exhibited similar estimated evolutionary rates. The estimated nucleotide substitution rates of Pol in HIV-1 subtypes B and C strains from Central America were 2.8–4.7 x10^-3^ and 1.5–2.3 x10^-3^substitutions per site per year, respectively. Taken together, we found that the Tat gene exhibited a lower substitution rate than structural genes like Env and Pol. However, the reason for this variation is yet to be revealed.

Our results of tMRCA for HIV-1 subtype B Tat in the United States was 1954–1955 with a variable rate of evolution obtained using the Bayesian approach. However, the tRMCA was found around 1967 using the strict molecular clock model. For external genes like Env, the tMRCA for HIV-1 subtype B in the United States and Western Europe was likely to be around the middle of the 1950s to the late 1960s [11,31,32] whereas in another showed that tMRCA for Env, Gag and Pol in Americas, Western Europe and Australia were found around 1960, 1962 and 1959, respectively [13]. According to the Pol gene-based analysis, the spread of subtype B in Americas occurred through a single introduction event in the Caribbean region around 1964 (1950–1967). It has also been speculated that subtype B-driven epidemics in those regions might have started during that time [12] which is in line with our current study findings. In case Asian countries such as in Thailand, the HIV-1 Env gene in subtype B strains were emerged in the mid-1980s [33]. However, in Korea, HIV-1 subtype B was emerged in the early 1960s [14]. Interestingly, we found two peaks for HIV-1 Tat in subtype B around those previously mentioned dates. On the other hand, we found that subtype C was emerged in Africa between 1960s and 1970s in HIV-1 Tat. However, for Env, Gag and Pol gene previous study found tMRCA were around 1950s, 1960s and mid 1950s, respectively [13]. Another study estimated the tMRCA for the HIV-1 subtype C Pol gene was estimated to be in the early 1970s; notably, these strains were introduced into the general population in several instances through heterosexual or mother-to-child transmission[10]. Moreover, our detected tMRCA of HIV-1 Tat for subtype C was found one decade later than the findings in a study conducted in Bangladesh, where they found large regional cluster of samples from Bangladesh, India, China and Myanmar, which dates back to the early 1960s in HIV-1 Gag [34]. Overall, the tMRCAs of HIV-1 Tat in subtypes B and C found in our study are in line with other genes of HIV-1.

In the current study, we observed that epidemiological trends proceeded relatively slowly. The time span of the HIV-1 epidemic often encompasses months or even years owing to slow inter-host transmission kinetics. Moreover, the chronic nature of the disease leads to the persistence of epidemics through diffusing the infection among vulnerable groups [35]. As described previously, the extent of transmissibility is influenced by the time period of infection dynamics as well as by the changing vulnerability of hosts after epidemics [36]. Infection from multiple genotypes in an individual host provides a low level of cross-immunity [37], which may lead to slow evolutionary kinetics of HIV-1 proteins. It is noteworthy to mention that using the appropriate phylodynamic approach is imperative when correlating an epidemic event to its underlying genetic changes. In this regard, we performed a coalescent tree analysis to determine the nucleotide diversity for each time unit within a given phylodynamic framework. We measured the viral dynamics using GMRF skyride analysis, which enabled us to measure the evolutionary dynamics of HIV-1 based on Tat.

Taken together, our study highlights the importance of examining the internal /regulatory genes of HIV-1 in gaining a more complete understanding of its evolutionary dynamics. To some extent, we could not properly compare our results with other published [11,14,31–33] results on other structural genes due to a lack of uniformity in the analyzed variables, including insufficient information about the substitution model and limited information about the protocol used. It is worthwhile to mention that the estimated time of origin or evolutionary rate often varies among the genes within a single virus strain. Moreover, homogeneity of an analyzed sequence dataset, methods of multiple sequence alignment, and different substitution models often generate some variations. Future studies can also be done to reveal the evolutionary changes in other subtypes than B and C. Despite those variations and shortcomings, to the best of our knowledge, our study, for the first time, reports the evolutionary dynamics of HIV-1 Tat. The study results therefore contribute to a better understanding of HIV-1 viral dynamics.

## Supporting Information

S1 Fig Phylogenetic trees of HIV-1 Tat. Figure A) Maximum likelihood (ML), and Figure B) Neighbor-joining phylogenetic tree of HIV-1 subtypes B and C sequences based on 300 nucleotide sites of Tat gene sequences generated through the Los Alamos database. GTR+I+Γ5nucleotide substitution model was employed with 1000 bootstrapped data sets using MEGA 6. The HIV-1 Subtypes B and C Tat sequences are shown in green and pink round bullets, respectively. SIV sequence, CPZ.US.85.US_Marilyn.AF103 was used as the out-group to root the tree (red square bullet).
(TIF)

S1 Table Accession numbers of the different subtypes of HIV-1 Tat used in this study obtained from the Los Alamos National Laboratory (LANL) HIV sequences. (XLSX)
