# Supplementary figures and images for: Evolutionary Dynamics of Tat in HIV-1 Subtypes B and C
Source: PLoS One. 2015 Jun 18;10(6):e0129896. doi: 10.1371/journal.pone.0129896 (PMC4472691; doi:10.1371/journal.pone.0129896)

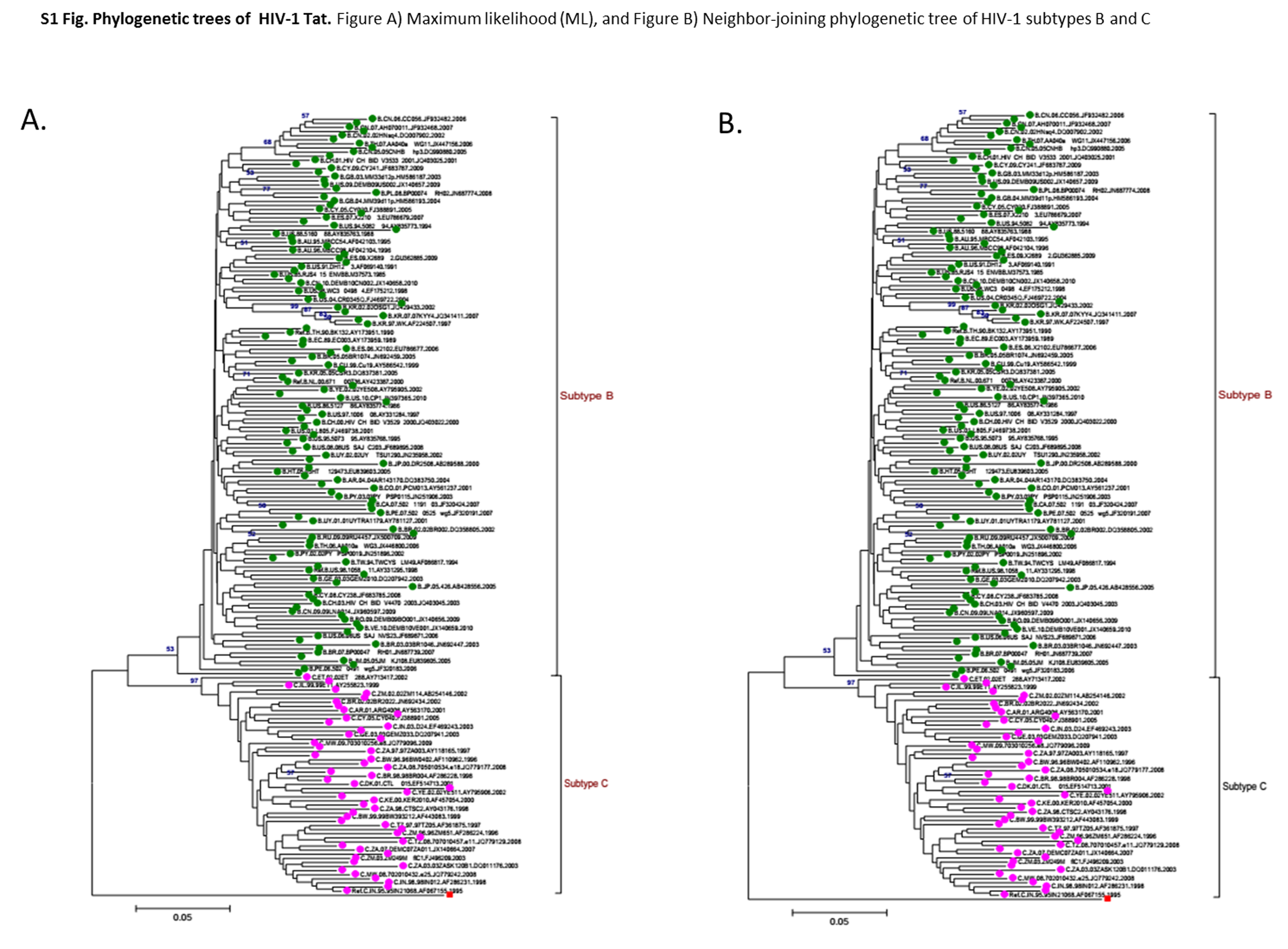

Supplement: S1 Fig — Figure A) Maximum likelihood (ML), and Figure B) Neighbor-joining phylogenetic tree of HIV-1 subtypes B and C sequences based on 300 nucleotide sites of Tat gene sequences generated through the Los Alamos database. GTR+I+Γ5nucleotide substitution model was employed with 1000 bootstrapped data sets using MEGA 6. The HIV-1 Subtypes B and C Tat sequences are shown in green and pink round bullets, respectively. SIV sequence, CPZ.US.85.US_Marilyn.AF103 was used as the out-group to root the tree (red square bullet). (TIF) [file pone.0129896.s001.tif]
